# A Cross-Sectional Analysis of Variation in Charges and Prices across California for Percutaneous Coronary Intervention

**DOI:** 10.1371/journal.pone.0103829

**Published:** 2014-08-04

**Authors:** Renee Y. Hsia, Yaa Akosa Antwi, Ellerie Weber, Julia Brownell Nath

**Affiliations:** 1 Department of Emergency Medicine, University of California San Francisco, San Francisco, California, United States of America; 2 Department of Economics, Indiana University Purdue University, Indianapolis, Indiana, United States of America; 3 School of Public Health, University of Texas, Houston, Texas, United States of America; University of California, San Francisco, United States of America

## Abstract

**Background:**

Though past studies have shown wide variation in aggregate hospital price indices and specific procedures, few have documented or explained such variation for distinct and common episodes of care.

**Objectives:**

We sought to examine the variability in charges for percutaneous coronary intervention (PCI) with a drug-eluting stent and without major complications (MS-DRG-247), and determine whether hospital and market characteristics influenced these charges.

**Methods:**

We conducted a cross-sectional analysis of adults admitted to California hospitals in 2011 for MS-DRG-247 using patient discharge data from the California Office of Statewide Health Planning and Development. We used a two-part linear regression model to first estimate hospital-specific charges adjusted for patient characteristics, and then examine whether the between-hospital variation in those estimated charges was explained by hospital and market characteristics.

**Results:**

Adjusted charges for the average California patient admitted for uncomplicated PCI ranged from $22,047 to $165,386 (median: $88,350) depending on which hospital the patient visited. Hospitals in areas with the highest cost of living, those in rural areas, and those with more Medicare patients had higher charges, while government-owned hospitals charged less. Overall, our model explained 43% of the variation in adjusted charges. Estimated discounted prices paid by private insurers ranged from $3,421 to $80,903 (median: $28,571).

**Conclusions:**

Charges and estimated discounted prices vary widely between hospitals for the average California patient undergoing PCI without major complications, a common and relatively homogeneous episode of care. Though observable hospital characteristics account for some of this variation, the majority remains unexplained.

## Introduction

Cost opacity for health care services has been proposed as one explanation for continually escalating health care costs. Most commercial markets guarantee customers relatively easy access to accurate information about the cost of services, thus enabling consumption to be tied to value. The health care marketplace, however, does not offer this transparency to patients or payers, a reality that leads to widespread variation in charges and prices [1], [2], [3], [4].

Past research attempting to explain the degree and sources of provider-level charge and price variation has generally focused on aggregate price indexes. [5], [6] However, creating indexes requires aggregating wide ranges of diagnoses and procedures. Charges for specific episodes of care, on the other hand, while not exactly the same should have less patient level variation and therefore more validity when evaluating between-hospital differences in charges and prices. [7] Further, the variation in charge for common episodes of care is of more use from a consumer perspective when deciding which hospital to visit for a specific complaint or procedure.

For instance, percutaneous coronary intervention (PCI) with a drug eluting stent is one of the leading surgical reasons for hospitalization in the United States [8] and one of the top ten contributors to healthcare costs, totaling over $18 billion in charges and over $5 billion in estimated costs in 2011. [9] Uncomplicated PCI with a drug eluting stent is a relatively standard procedure, involving only minor variation between patients. As a result, hospital charges for uncomplicated PCI with a drug-eluting stent (Medical Severity Diagnosis Related Group [MS-DRG] 247) should be fairly uniform in a competitive market, and any observed variation in charges is unlikely to result from different treatment choices. For this reason, uncomplicated PCI is an interesting condition for which to isolate and analyze hospital-level variation in charges and discounted prices.

We therefore conducted a cross-sectional analysis of patients admitted to California hospitals for PCI with a drug eluting stent, without major complications (MS-DRG 247) in 2011. Using a two-part linear regression model, we first predicted charges at each hospital after adjusting for patient characteristics. We then assessed the variation in these adjusted charges for the average California patient at each hospital, and used them as the dependent variable in a second regression, in which we assessed whether hospital and market-level factors could explain some of the between-hospital variation in charges. Finally, we calculated the variation in estimated discounted prices paid by private insurers. We hypothesized that variation in charges for PCI would be small after accounting for hospital and market characteristics, and that numerous institutional covariates would be associated with hospital charges for uncomplicated PCI.

## Methods

### Data Sources

To capture admissions for uncomplicated PCI, we used the 2011 publicly available Patient Discharge Database from the California Office of Statewide Health Planning and Development (OSHPD). This dataset captures demographic and clinical data as well as reported charges for all admissions to non-federal hospitals in California, excluding those operated by Kaiser Permanente (a large managed care organization in California), which are not required to report charges to OSHPD. In this public dataset, OSHPD masks selective patient information pursuant to the California Health Data and Advisory Council Consolidation Act. Because we used a public data source that was masked for identifiers, our study was exempt from review by the Committee on Human Research at the University of California, San Francisco.

To capture hospital-level factors including each hospital’s ownership, teaching status, rural/urban status, and number of licensed beds, we used 2011 hospital financial and utilization files available from OSHPD. [10] We then used the Area Resource Files from the Health Resources and Services Administration to measure each hospital market’s uninsured population and poverty rates. [11] Finally, we used the Impact Files from the Centers for Medicare & Medicaid Services (CMS) to capture each hospital’s wage index (cost of living) and case-mix index [12].

### Sample Selection

**Figure 1:**
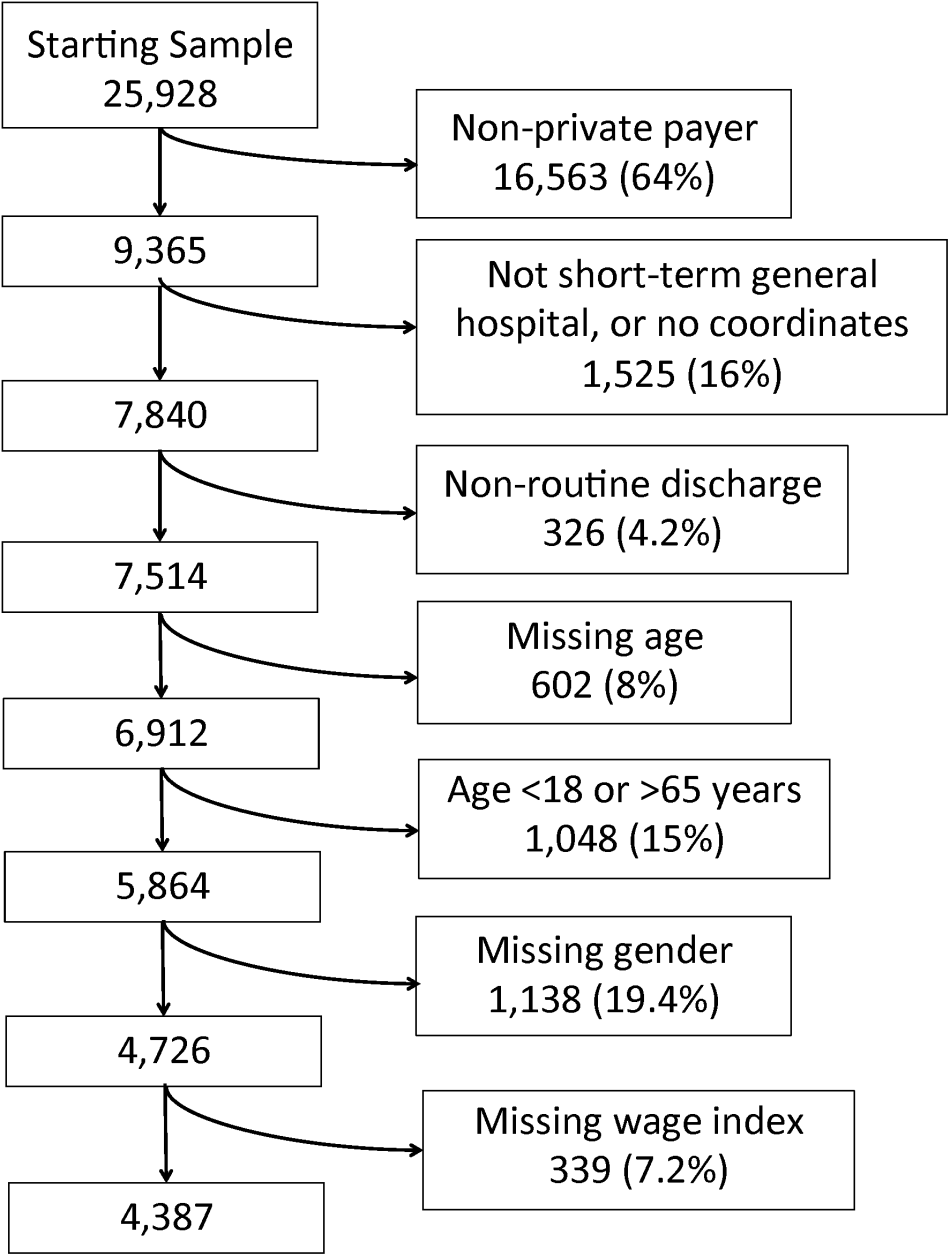
Sample Selection. Flow chart of exclusions from the original starting sample of all adult (≥18 years old) patients admitted for MS-DRG-247 leading to the final 4,387 patients studied. Missing variables generally referred to masked items in the public dataset used to protect the identity of patients. “No coordinates” indicates an inability to locate the hospital.

We included data on all adult patients (18–64 years old) admitted to a general acute care hospital for MS-DRG 247 – PCI with a drug eluting stent, without major complications. We further limited our sample to privately insured patients, as the discount factor we later use to estimate discounted price only applies to them. In an effort to maintain a homogeneous sample, we excluded patients who died in the hospital and those who did not have a routine discharge home. Patients with invalid charges, charges exceeding the cell size limit, and those receiving charity care were also excluded from the analysis. See Figure 1 for a full description of our exclusions.

### Outcomes

Our primary outcome was total hospital charges for an admission for MS-DRG-247. These charges represent the total amount billed by the hospital to the patient or his insurance for the episode of care, excluding physician fees. Charges are calculated using the full, established rates before any adjustments or pre-payments.

As a secondary outcome, we examined estimated discounted prices, which represent the amount hospitals actually receive for the services they provide. We calculated this measure by multiplying the charge by the hospital’s average discount rate for all privately insured patients, as done in previous work. [6], [13] The average discount rate was calculated from the 2011 OSHPD financial files as follows: (gross inpatient revenue + gross outpatient revenue – contractual adjustments)/(gross inpatient revenue + gross outpatient revenue) [13].

### Patient Level Predictors

In adjusting charges for patient characteristics, we considered patient age (<40, 40–44, 45–49, 50–54, 55–59, and 60–64 years old), gender, Charlson comorbidity scores, Elixhauser comorbidities (hypertension, diabetes without chronic complications, diabetes with chronic complications, peripheral vascular disease, chronic pulmonary disease, hypothyroidism, renal failure, anemia, depression, and obesity), [14] insurance coverage (Knox-Keene/Medi-Cal Organized Health System, other managed care, or traditional private coverage), and length of stay as patient-level predictors of charge. Length of stay is a right-skewed variable, and to control for this, we log-transformed each value (length of stay+1) [15].

### Hospital and Market-Level Predictors

To look at hospital-level influences on charges, we included variables for hospital ownership (for profit, non-profit, government), teaching status, urban or rural location, volume (number of licensed beds), patient payer mix (% Medicare, % Medicaid), wage index (a measure of cost of living), and case-mix severity. We also included two facility-level inpatient quality indicators from the Agency for Healthcare Research and Quality: acute myocardial infarction (AMI) mortality rate, and heart failure mortality rate [16].

We further included the percent of the population in the hospital’s county that is uninsured, percent of the county in poverty, and the Herfindahl-Hirschman Index (HHI) of the catchment area as market-level characteristics related to hospital charges. The HHI is widely used as a measure of the level of competition in an industry, and is defined as the sum of squares of the market shares of all hospitals within the market, here defined as all zip codes the hospital draws patients from. [17] It can range from 0 to 10,000 (using whole percentages), and a higher index signifies less competition. We calculated market shares directly from our patient discharge data. We also accounted for hospitals’ membership in systems by calculating system-wide HHI because hospital system membership can influence price setting [6].

### Statistical Analysis

We used a two-part regression model for our analysis to specifically assess between-hospital variation in charges for uncomplicated PCI. First, we regressed the log of raw hospital charges on the aforementioned patient clinical and demographic characteristics that could affect the level of services provided. Dummy variables for each hospital were included as fixed effects. This model was used to predict the charge per average length of stay at each hospital for the average California patient with uncomplicated PCI. We then descriptively analyzed these predicted charges to assess the degree of between-hospital variation in charges for uncomplicated PCI that is not driven by differences in observable patient demographics or comorbidities.

In our second regression, we regressed the log of these predicted charges on the hospital and market characteristics mentioned above. The exponentiated coefficients from this model tell us if and how hospital and market characteristics significantly predict charges, as described in previous literature. [6], [13], [18] This second regression also tells us what proportion of the variation we observe between hospitals is explained by the observable hospital and market characteristics in the model. All analyses were completed using STATA version 11.0 (College Station, TX).

## Results

Our final sample included 4,387 privately insured patients admitted to one of 124 California hospitals for PCI with a drug eluting stent and without major complications (MS-DRG-247) in 2011. The sample was 80% male, and 60% were between the ages of 55 and 64. (Table 1). For 76% of the sample their length of stay was shorter than 3 days, and 50% had a Charlson index of 1, indicating serious but relatively simple admissions. Many patients had comorbidities; 64% had hypertension, and almost 30% had diabetes. Of the 124 hospitals, 71% were not-for-profit, 99% were urban, and 15% were teaching hospitals (Table 2). Market characteristics showed some variability; 60% of hospitals were in markets with low cost of living, and 31% were in markets with a low degree of competition.

**Table 1:** Characteristics of patients in the study sample (n = 4,387).

Age categories	N	%
<40 years	86	2.0%
40–44	253	5.8%
45–49	498	11.4%
50–54	917	20.9%
55–59	1,223	27.9%
60–64	1,410	32.1%
**Sex**		
Male	3,498	79.7%
Female	889	20.3%
**Private Insurance Type**		
Managed Care-Knox Keene	1,952	44.5%
Managed Care-Other	2,070	47.2%
Traditional Coverage	365	8.3%
**Charlson Index**		
0	1,031	23.5%
1	2,199	50.1%
2	1,157	26.4%
**Length of Stay**		
<3 days	3,329	75.9%
3–6 days	1,032	23.5%
>6 days	26	0.6%
**Elixhauser Comorbidities**		
Hypertension	2,818	64.2%
Diabetes w/o chronic complications	1,152	26.3%
Diabetes w/chronic complications	130	3.0%
Peripheral vascular disease	160	3.7%
Chronic pulmonary disease	313	7.1%
Hypothyroidism	246	5.6%
Renal failure	141	3.2%
Anemia	147	3.4%
Obesity	746	17.0%
Depression	178	4.1%

**Table 2:** Characteristics of California hospitals in sample (n = 124).

Ownership	N	%	
Government	11	8.9%	
Non-profit	89	71.8%	
For-profit	24	19.4%	
**Location**			
Urban	123	99.2%	
Rural	1	0.8%	
**Teaching Status**			
Yes	19	15.3%	
No	105	84.7%	
**Wage Index (tertiles)**	**N**	**Mean**	**SD**
Low	75	1.20	0.007
Medium	8	1.22	0.011
High	41	1.54	0.108
**Herfindal-Hirschman Index (tertiles)**			
Low	42	1304	476
Medium	43	3212	708
High	39	6475	2144
**Casemix (severity – tertiles)**			
Low	42	1.52	0.08
Medium	41	1.68	0.04
High	41	1.88	0.16
**% Without Insurance**	124	18.1%	3.2%
**% Below Federal Poverty Line**	124	12.5%	3.1%
**Licensed Beds**	124	372	174
**% Medicare**	124	40.8%	11.2%
**% Medicaid**	124	23.3%	13.6%

Looking first at raw charges for an admission for PCI without major complications, we found a median raw charge of $97,589. These raw charges varied substantially, ranging from $20,056 to $195,245. We then adjusted the raw charges for the patient’s characteristics to predict adjusted charges for the average patient with a hospital stay for PCI with a drug eluting stent and without major complications at each hospital (see Table S1 for the results of this first regression). Our predicted charges ranged from $22,047 to $165,386 (median $88,350) depending on which hospital the patient visited.

Many hospital and market attributes were significantly correlated with these adjusted charges for PCI in the average patient (Table 3). Our model revealed that government-owned hospitals charged 28% less than not-for-profit hospitals, while rural hospitals charged 36% more than urban hospitals. Hospitals located in areas with the highest cost of living (wage index) had 39% higher adjusted charges than those in areas with the lowest costs of living, and for each one percent increase in the proportion of a hospital’s patients covered by Medicare, charges for PCI without major complications increased by 0.7%. Overall, our model explained 43% of the variation in adjusted charges (R^2^ = 0.4308).

**Table 3:** The impact of hospital and market characteristics on adjusted charges.

	% Increase in chargesfor each unit changein predictor	95% CIlower bound	95% CIupper bound	p-value
**HOSPITAL-LEVEL CHARACTERISTICS**				
**Ownership**				
Government	−28.1%	−44.6%	−6.8%	0.013
Non-profit	ref			
For-profit	6.2%	−6.8%	22.1%	0.371
**Teaching Status**				
Yes	−10.4%	−29.5%	12.7%	0.346
No	ref			
**MSA**				
Urban	ref			
Rural	36.3%	13.9%	63.2%	0.001
**Volume**				
No. of licensed beds	0.0%	0.0%	0.0%	0.118
**Patient Mix**				
% Medicare	0.7%	0.0%	1.4%	0.048
% Medicaid	0.5%	−0.1%	1.1%	0.136
**Casemix (severity)**				
Medium	13.9%	−0.4%	31.0%	0.057
High	12.7%	−2.1%	28.9%	0.097
**Wage Index**				
Medium	28.3%	−15.5%	94.8%	0.239
High	38.5%	15.7%	65.7%	0.001
**Quality Indicators**				
AMI mortality rate (%)	1.5%	−1.7%	4.7%	0.365
Heart failure mortality rate (%)	5.2%	−2.4%	10.9%	0.061
**MARKET-LEVEL CHARACTERISTICS**				
**% Without Health Insurance**	−0.1%	−2.4%	2.7%	0.915
**% Below Federal Poverty Line**	−1.7%	−5.6%	2.3%	0.404
**Herfindal-Hirschman Index (System-wide)**				
Medium	−2.1%	−20.5%	20.7%	0.842
High	−18.7%	−34.5%	0.9%	0.06

Legend: In this second step of our two-part regression, we regressed hospital and market characteristics on the log of the adjusted average charges per length of stay at each hospital generated from the first regression. The effects displayed here represent the impact the variable in question had on the hospital’s charge for the average California patient. They are calculated as the difference between the exponentiated coefficients from the model and one, to show percent change.

**Figure 2:**
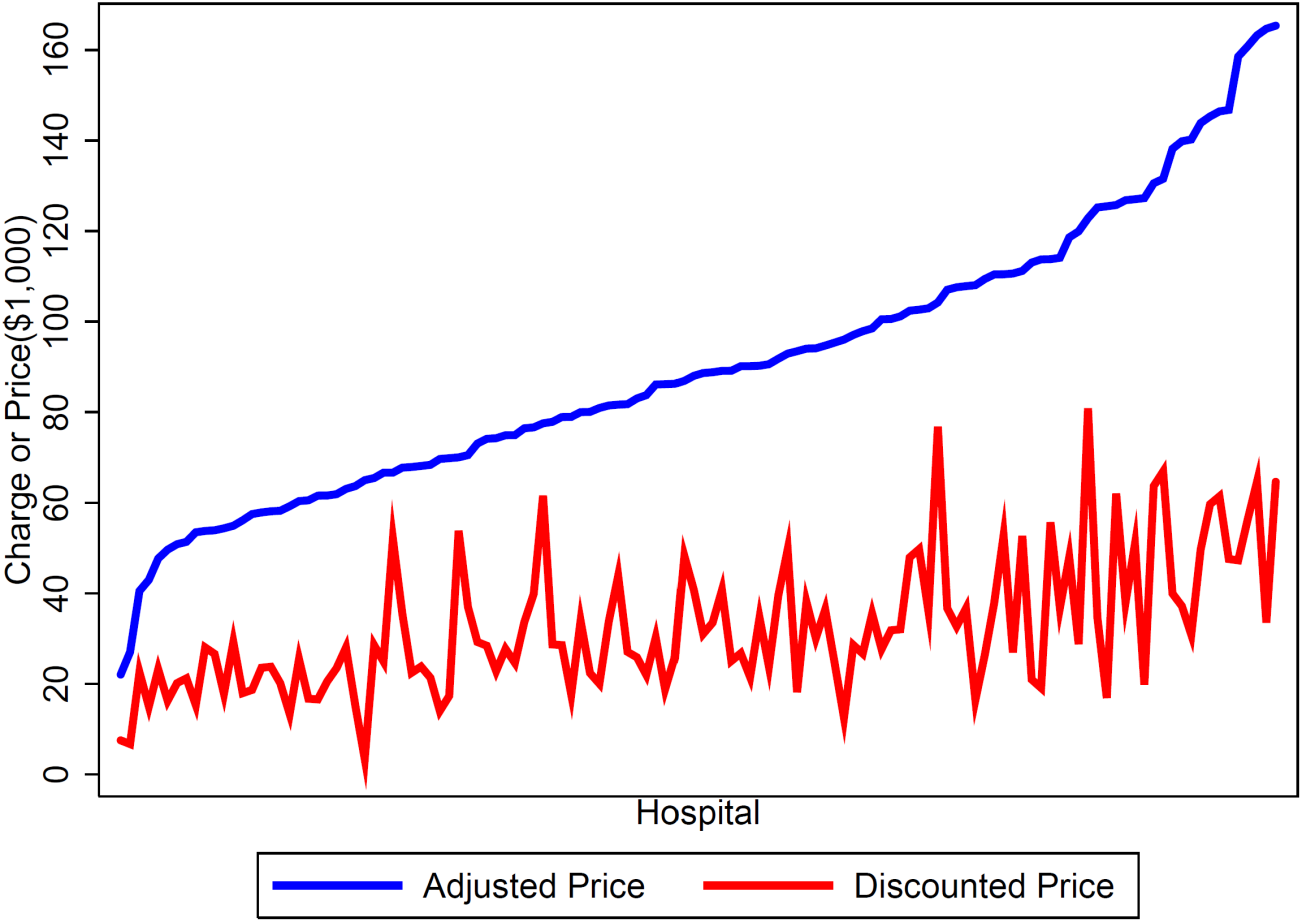
Discounted prices versus adjusted charges, by hospital. Hospitals are placed in order along the x-axis by charge for the average patient admitted for MS-DRG 247 (blue). The corresponding discounted price (estimated paid by a private insurer) is represented in red.

Finally, we used the product of the predicted charges and average estimated discount rates for each hospital to estimate what price a private insurer would actually pay for PCI without major complications. The calculated discounted prices for the average patient at each hospital ranged from $3,421 to $80,903, with a median discounted price of $28,571 – less than one third of the median adjusted charge. Figure 2 shows the adjusted charges and corresponding discounted prices for each hospital, demonstrating that while the measures are correlated, charge is not a perfect predictor of price.

## Discussion

Our findings show that for the average California patient admitted for PCI with a drug eluting stent and without major complications in 2011, charges varied from $22,047 to $165,386 depending on the hospital he visited. This range only represents between-hospital variation in charges, as the variation in raw charges stemming from observable patient characteristics was removed in our first-stage regression. We found that hospital and market-level characteristics did help explain some of this between-hospital variation in charges for uncomplicated PCI. For instance, government hospitals charged less than not-for-profit hospitals. Hospitals in markets with high costs of living charged more, as did rural hospitals and hospitals with higher proportions of Medicare patients. These findings are generally aligned with those of previous literature studying broad price indices [6], [18].

However, our model explained only 43% of the variation in charges between hospitals, and relatively few predictors were significant. The large proportion of variation that remained unexplained could be due either to unobservable hospital or market level factors, or to entirely random differences in hospital charge levels.

Past literature indicates that much of the unexplained variation we observe is likely unrelated to cost or hospital and market characteristics. A MedPAC-funded national survey of hospital financial administrators found that many chargemasters, from which DRG charges are aggregated, are based on historical prices that were calculated before costs of any given service could be accurately estimated. [19] When setting and maintaining their chargemasters today, most hospitals surveyed were concerned with meeting regulations and maintaining their overall bottom line, while only one third of hospitals interviewed reported any concern over costs. [19] This is not surprising, as third party payments from insurers are not based on costs, providing no incentive for hospitals to consider costs when setting their charges. [20], [21] Simplistic “updates” that raise all charges by a uniform percentage exacerbate the problem, as they aim to maintain the overall solvency of the hospital and result in differential profitability of services. [19], [21], [22] These practices prevent a substantial relationship between charges and costs. Without this relationship, which is present in most other competitive industries, there is no basis to limit variation in charges between different hospitals. Our results confirm the presence of unexplainable variation, and thus support the documented absence of systematic charge setting in the chargemaster system.

However inexplicable charges may be, they nevertheless can have a tangible impact on patients and hospitals. Privately insured patients seeking care out of network and the 22% of American adults aged 19–64 who are uninsured may be billed the full charge of their care. [23] Most charges are so high that patients cannot pay them in full, which, without charity care or sliding-scale income adjustment, can result in bad debt. [24], [25] In part as a result of this system, 57% of all American bankruptcies are related to medical bills. [26] California’s Fair Pricing Act of 2006 has significantly reduced the hardship of high hospital bills on uninsured patients, and now 97% of California hospitals provide free care to uninsured patients with incomes below 100% of the federal poverty line. [27] The Affordable Care Act attempts to implement similar fair pricing strategies nationwide, but the provision applies only to non-profit hospitals and only specifies the need to provide “financial assistance” for the uninsured, leaving significant room for hospital interpretation and difficult enforcement of meaningful changes [27].

Hospitals use charges regularly in calculating their uncompensated care costs; 18–20% use the difference between charges and payments by private insurers, and 50% use the difference between charges and payments from the uninsured in these calculations. [28] These amounts are then used to determine a hospital’s not-for-profit, and hence tax-exempt status. [29] In addition, Medicare sets their relative DRG weights and identifies qualifying outlier payments using the product of charges and cost center level cost to charge ratios. [30], [31], [32], [33] Finally, many private insurers still base their fee-for-service reimbursements off discounted charges, and even insurers using prospective payment systems sometimes use charges in benchmarking those payments [21], [25].

In our secondary analysis, we found that estimated discounted prices were, on average, approximately one third of the predicted charge. They also showed significant variation across hospitals (range: $3,421–$80,903). These discounts reflect the market power of private insurance companies to negotiate discounted prices. [34], [35] For reference, CMS estimated that the average cost for MS-DRG 247 was $13,014 per visit in 2012. [36] For consumers who are billed their full charges, this difference between the discounted price and actual charges has profound financial implications. [37] This form of cost shifting actually penalizes those individual consumers who have the least power in the system and the lowest ability to pay [38].

### Limitations

Our results should be interpreted in light of three major limitations. First, because we used a DRG and not line-item services (which were unavailable in our data) to classify an episode of care for PCI with a drug eluting stent and without major complications, it is likely that each patient had slightly different intensity of utilization during their stay. Though we attempted to minimize the impact of this limitation through our first regression (that controlled for observable patient factors correlated with intensity), there were likely unobservable confounding patient characteristics that could have explained more of the variation in charges at the individual level. However, as we used hospital-level fixed effects in our first model, these differences should not affect our second-step results unless the unobservable patient characteristics are correlated with the hospital characteristics that we included as regressors.

Second, the OSHPD data reports discount rates for privately insured patients on an aggregate, hospital-level basis. However, private payer reimbursements likely vary by insurer, DRG, and department. We therefore recognize that our estimates of discounted final prices are measured with error, and thus we focus our regression analysis on charges, which are reported more precisely in our data. However, past analyses have found that insurers often broadly apply discount rates to wide ranges of services, as the negotiated rates are aimed to maintain institutional solvency. [39] In addition, there is significant precedent for applying ratios to charges at the aggregate level; for example cost-to-charge ratios applied directly to charges are used at an aggregate level by CMS to estimate costs, and have been shown to be imperfect but generally acceptable proxies for actual cost [40].

Finally, it is important to note that our study is limited to California, and though our results provide an interesting case study of charge variation in a large and diverse state, they cannot be generalized to the entire nation.

## Conclusions

In 2011, the average California patient with a hospital stay for PCI with a drug eluting stent who did not experience any major complications could be charged between $22,047 and $165,386 (median $88,350) depending on which of 124 hospitals he visited. Discounted prices paid by private insurers were, on average, approximately one-third of the charges. Hospital ownership, share of patients insured by Medicare, cost of living, and rural location were correlated with charge rates. However, observable hospital and market-level factors explained only 43% of the between-hospital variation in charges. These findings demonstrate the wide and largely unexplained variation in charges and prices for a common and relatively homogeneous episode of care.

## Supporting Information

Table S1 **The impact of patient characteristics on raw charges.** In this first step of our two-part regression model, we regressed multiple patient demographic and clinical characteristics listed above, along with hospital fixed effects, on the log of raw charges. The percent impact of each covariate on charges shown here represent the difference between the exponentiated coefficients from the model and one, to indicate percent change. This regression not only generated the impact of patient characteristics on charges, but also was used to estimate the adjusted charge per average length of stay at each hospital.
(DOCX)
