# Supplementary material for: A Cross-Sectional Analysis of Variation in Charges and Prices across California for Percutaneous Coronary Intervention
Source: PLoS One. 2014 Aug 4;9(8):e103829. doi: 10.1371/journal.pone.0103829 (PMC4121236; doi:10.1371/journal.pone.0103829)
Supplement: Table S1 — The impact of patient characteristics on raw charges. In this first step of our two-part regression model, we regressed multiple patient demographic and clinical characteristics listed above, along with hospital fixed effects, on the log of raw charges. The percent impact of each covariate on charges shown here represent the difference between the exponentiated coefficients from the model and one, to indicate percent change. This regression not only generated the impact of patient characteristics on charges, but also was used to estimate the adjusted charge per average length of stay at each hospital. (DOCX) [file pone.0103829.s001.docx]

|  | % Increase in charges for each unit change in predictor | 95% CI lower bound | 95% CI upper bound | p-value |
| --- | --- | --- | --- | --- |
| **Charlson index** | -3.8% | -6.8% | -0.8% | 0.014 |
| **Log(length of stay)** | 61.6% | 54.8% | 68.7% | <0.001 |
| **Female** | -2.9% | -4.8% | -0.9% | 0.005 |
| **Age group** |  |  |  |  |
| <40 years | ref |  |  |  |
| 40-44 years | 5.4% | -5.1% | 17.0% | 0.323 |
| 45-49 years | 7.5% | -3.3% | 19.4% | 0.182 |
| 50-54 years | 6.3% | -4.3% | 18.2% | 0.251 |
| 55-59 years | 5.8% | -4.7% | 17.5% | 0.287 |
| 60-64 years | 5.4% | -4.9% | 16.8% | 0.310 |
| **Payer** |  |  |  |  |
| Managed Care - Knox Keene | ref |  |  |  |
| Managed Care - Other | 1.3% | -1.9% | 4.7% | 0.424 |
| Traditional Coverage | -0.5% | -4.2% | 3.4% | 0.798 |
| **Elixhauser Comorbidities** |  |  |  |  |
| Hypertension | -0.5% | -2.6% | 1.6% | 0.622 |
| Diabetes w/o chronic complications | -0.5% | -2.4% | 1.5% | 0.624 |
| Diabetes w/ chronic complications | 1.6% | -2.1% | 5.4% | 0.390 |
| Peripheral vascular disease | 3.0% | -1.9% | 8.1% | 0.230 |
| Chronic pulmonary disease | -0.8% | -3.8% | 2.4% | 0.637 |
| Hypothyroidism | 0.9% | -1.6% | 3.5% | 0.473 |
| Renal Failure | -0.9% | -5.4% | 3.8% | 0.691 |
| Anemia | 2.8% | -1.4% | 7.4% | 0.197 |
| Depression | 0.5% | -2.8% | 3.9% | 0.751 |
| Obesity | 1.0% | -1.2% | 3.4% | 0.353 |
